# Integrated Analysis of the Clinical and Molecular Characteristics of IDH Wild-Type Gliomas in the Chinese Glioma Genome Atlas

**DOI:** 10.3389/fonc.2021.696214

**Published:** 2021-07-07

**Authors:** Peng Wang, Yanwei Liu, Lin Zhi, Xiaoguang Qiu

**Affiliations:** ^1^ Department of Radiation Oncology, Beijing Tiantan Hospital, Capital Medical University, Beijing, China; ^2^ Department of Molecular Neuropathology, Beijing Neurosurgery Institute, Capital Medical University, Beijing, China

**Keywords:** Lower-grade glioma, Glioblastoma, IDH wild-type, molecular pathology, whole exome sequencing

## Abstract

**Purpose:**

Current studies and guidelines suggest that the biobehavior of IDH-wild type (IDH-wt) lower-grade glioma (LGG, WHO II-III) is similar to IDH-wt glioblastoma (GBM). However, differences in their clinical and molecular characteristics have not been reported. This study aimed to analyze the clinical and genetic information of gliomas with IDH-wt.

**Methods:**

389 patients with IDH-wt were enrolled in the study (LGG=165, GBM=224), and their clinical and genetic information was collected from the Chinese Glioma Genome Atlas (CGGA). We conducted an analysis of this information between the two groups of patients and drew conclusions thereof.

**Results:**

The median age of the LGG patients was 42 (18–74) years, whereas that of the GBM patients was 51 (18–79) years (*P* < 0.010). GBM patients were more likely to undergo total resection (*P* = 0.018) and had fewer epileptic seizure symptoms (*P* < 0.001). The median overall survival (OS) was 55 months for the LGG patients and only 14.83 months for the GBM patients (*P* < 0.01). The median progression-free survival (PFS) was 44 months for the LGG patients and only 9.767 months for the GBM patients (*P* < 0.001). GBM patients were more prone to PETN mutations (*P* = 0.010). Transcriptome analysis showed that the differentially expressed genes in LGG patients were mainly enriched in metabolic pathways and pathways in cancer and in the function of signal transduction and positive regulation of GTPase activity, whereas in GBM patients, they were mainly enriched in the PI3K-Akt signaling pathway and in the functions of apoptotic process and oxidation-reduction process.

**Conclusions:**

Our data indicate that these two groups of patients should be re-evaluated and treated differently, despite both having IDH wild type.

## Introduction

Gliomas are the most common and lethal type of primary malignant central nervous system (CNS) tumor, with an extremely poor prognosis. They comprise approximately 30% of all brain tumors and 80% of all malignant brain tumors (1). According to the World Health Organization (WHO) classification of tumors of the CNS, malignant adult diffuse gliomas are classified into grades II to IV based on their histologic features. In the 2016 edition of the classification, gliomas were subdivided into more subtypes based on molecular features, such as 1p/19q codeletion and IDH mutational status (2–4). With the addition of molecular pathology to the diagnosis of glioma by the WHO in 2016, the classification of glioma has undergone new changes, providing a new basis for the prediction of patient treatment and prognosis, thus improving the accuracy of treatment (5, 6). With the newly proposed molecular pathology-based diagnosis, IDH, as a very important classification standard, has been widely used in the classification and diagnosis of glioma (7). According to Yan et al.’s research, more than 80% of LGGs harbor an IDH mutation, including diffuse astrocytoma (grade II, 90%), pleomorphic xanthoastrocytoma (grade II, 14%), and anaplastic astrocytoma (grade III, 73%), whereas only 5% of primary GBMs contain IDH mutations (2, 3, 8).

Current studies have mainly focused on a comparison between IDH mutant-type LGG and GBM. Few studies have been conducted on glioma with IDH-wt. According to the latest studies by the RTOG 9802 and Kosuke et al., the prognosis of IDH-wt LGG is substantially poor, with a median OS and PFS of 22.8 and 8.4 months, respectively, close to those of GBM (9, 10). In addition, in terms of molecular genetic background, IDH-wt LGG and GBM are similar, and some researchers believe that IDH-wt GBM may develop from IDH-wt LGG (11). In addition, in the third cIMPACT-NOW report, the committee recommended reclassifying IDH1/2 wild-type diffuse lower-grade gliomas of WHO grade II and III (LGG) as diffuse astrocytic glioma, IDH1/2-wt with molecular features of glioblastoma (12). Moreover, NCCN guidelines recommend that IDH wild-type LGG be treated using therapy typical for GBM (13). However, according to the abovementioned reports and the latest literature, as well as our data, we found that IDH-wt LGG and IDH-wt GBM may have differences in their prognoses and molecular features, such as TERT promoter mutation and EGFR amplification, which may lead to differences in treatment (12, 14).

Our study explored the differences between the two types of glioma by comparing the clinical and genetic information of 389 patients, which will provide a theoretical basis for better distinguishing the two types of patients and future precision medicine.

## Methods

### Patients

We screened 850 IDH wild-type glioma patients from 2546 glioma patients in the Chinese Glioma Genome Atlas (CGGA) database (http://cgga.org.cn/), and a further 389 patients were obtained according to the following conditions: they have complete clinical information, older than 18 years, newly diagnosed, and received the standard treatment regimen. All patients were followed up until May 2019 or death, after they received the first operation. Their clinical characteristics, such as age, sex, resection range, survival, and chemotherapy regimen, and molecular pathological information, such as 1p/19q co-deletion, MGMT methylation status, and PTEN mutational status, were collected for subsequent analysis. We also selected 140 of the 389 patients to collect their mRNA sequencing (mRNA-seq) data. All patients received the treatment regimen based on the NCCN guideline and received the MRI examination within 24 h after the operation to assess the extent of resection (15). All research performed was approved by the Tiantan Hospital Institutional Review Board (IRB). All the subjects were diagnosed with glioma by consensus, according to central pathology reviews by independent board-certified neuropathologists and further graded based on the 2007/2016 WHO classification. Written informed consent was obtained from all patients.

### Identification of Molecular Alterations

IDH1/2 (IDH) mutational status and the methylation status of the O6-methylguanine-DNA methyl-transferase (MGMT) promoter were detected by pyrosequencing. We predicted 1p/19q codeletion status by FISH. To determine the mutation status of P53 and PTEN, we used an immunohistochemical method (16–19).

### Data Analysis

To identify the gene sets related to particular biological processes present in IDH-wt LGG and GBM, we screened 140 patients from 398 patients (61LGG, 79GBM), and collected their WEseq data, then gene expression profiling and gene set enrichment analysis (GSEA) were performed as described previously (20). The analysis we conducted was based on that found on the Database for Annotation, Visualization, and Integrated Discovery (DAVID) website (21, 22). Survival distributions were estimated with Kaplan-Meier survival analysis, and log-rank analysis was used to assess the significance of differences between stratified survival groups using GraphPad Prism 8.0 statistical software. The χ^2^ test was performed by SPSS software (IBM SPSS Statistics 24). We found the gene sequencing data of the enrolled patients through the CGGA database, calculated the mean value of each gene, and then used unpaired *t* test grouping to select genes of interest in IDH-wt LGG and GBM. We used R Studio to perform supervised cluster analysis on the screened differential genes to identify whether they were different from each other.

## Results

### Clinical Information Analysis

In this study, we enrolled 165 LGG patients and 224 GBM patients. In terms of clinical information, LGG cohort has 99 male and 66 female, GBM cohort has 146 male and 78 female patients. In terms of clinical information, we found that the median age of the IDH wild-type LGG and GBM patients was 42 years (range, 18–74 years; primarily 40–47 years, with a median age of 42 years) and 51 years (range, 18–79 years; primarily 47–53 years; with a median age of 51 years), respectively (*P* < 0.010). 135 (60.3%) IDH-wt GBM patients and 77 (46.7%) LGG patients underwent total resection (*P* = 0.018). Epileptic symptoms were noted for 50 (22.3%) IDH-wt GBM patients and 82 (49.7%) IDH-wt LGG patients (*P* < 0.001). There was no significant difference in the extent of invasion (multilobe LGG, 56 patients (33.9%); multilobe GBM, 74 patients (33%); *P* = 0.264) or sex (*P* = 0.296) between the two groups of patients. Data for all of the above patients are shown in **Table 1**. As to therapy, 134 LGG patients (81.2%) received radiotherapy and 190 GBM patients (84.8%) received radiotherapy (*P* = 0.072). 80 LGG patients (48.5%) received chemotherapy and 159 GBM patients (71.0%) received chemotherapy (*P* < 0.001).

**Table 1:** Characteristics of clinical and molecular in this study.

Characteristics	II-III grade (IDH wild-type)	Primary GBM (IDH wild-type)	P value
Total	165 (42.4%)	224 (56%)	NA
Age	42 (18-74)	51 (18-79)	<0.01
Gender			0.296
Male	99 (60.0%)	146 (65.2%)	
Female	66 (40.0%)	78 (34.8)	
Resection			0.018
Total resection	77 (46.7%)	135 (60.3%)	
Subtotal resection	74 (44.8%)	78 (34.8%)	
NA	14 (8.5%)	11 (4.9%)	
Tumor location			0.264
Multi lobe	56 (33.9%)	74 (33.0%)	
Single lobe	78 (47.3%)	79 (35.3%)	
NA	31 (18.8%)	71 (31.7%)	
Symptom			<0.001
Seizure	82 (49.7%)	50 (22.3%)	
No seizure	69 (41.8%)	153 (68.3%)	
NA	14 (8.5%)	21 (9.4%)	
Radiotherapy			0.072
Yes	134 (81.2%)	190 (84.8%)	
No	27 (16.4%)	22 (9.8%)	
NA	4 (2.4%)	12 (5.4%)	
Chemotherapy			<0.001
Yes	80 (48.5%)	159 (71.0%)	
No	78 (47.3%)	56 (25.0%)	
NA	7 (4.2%)	9 (4%)	
Median PFS (Mo)	44	9.767	<0.001
Median OS (Mo)	55	14.83	<0.001
1p/19q codeletion			NA
Yes	0 (0%)	0 (0%)	
No	51 (30.9%)	106 (47.3%)	
NA	114 (69.1%)	118 (52.7%)	
MGMT methylation			0.520
Yes	25 (15.2%)	69 (30.8%)	
No	42 (25.5%)	140 (62.5%)	
NA	98 (59.4%)	15 (6.7%)	
PETN			0.010
Yes (wild)	88 (53.3%)	79 (35.3%)	
No (mutant)	8 (5.0%)	26 (11.6%)	
NA	69 (41.7%)	119 (53.1%)	
P53			0.518
Yes (wild)	87 (52.7%)	88 (39.3%)	
No (mutant)	13 (7.9%)	17 (7.6%)	
NA	65 (39.4%)	119 (53.1%)	
TERT			0.991
Mutant	12 (7.3%)	22 (9.8%)	
Wild	17 (10.3%)	31 (13.8%)	
NA	136 (82.4%)	171 (76.4%)	
EGFR			0.204
Amplification	7 (4.2%)	14 (6.3%)	
Wild	125 (75.8%)	138 (61.6%)	
NA	33 (20%)	72 (32.1%)	

### Molecular Pathological Characteristics

Among the patient information we collected, 1p/19q codeletion occurred in none of the IDH-wt GBM patients (0%) or IDH-wt LGG patients (0%). About 5% LGG patients have PTEN mutation versus 11.6% GBM patients (*P* = 0.010). However, in our data, there was no significant difference in MGMT methylation status (15.2% LGG has methylation and 30.8% GBM has methylation, *P* = 0.520), P53 mutational status (7.9% LGG was mutant, 7.6% GBM was mutant, *P* = 0.518), TERT promoter mutations (7.3% LGG was mutant, 9.8% GBM was mutant, *P* = 0.991), and EGFR amplification (4.2% LGG was amplification and 6.3% GBM was amplification, *P*=0.204) between the two groups of patients (**Table 1**).

### Survival Analysis

**Figure 1:**
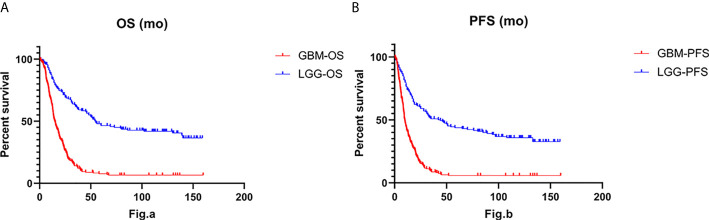
The Log-rank test and Gehan-Breslow-Wilcoxon test for overall survival (OS) **(A)** and progression-free survival (PFS) **(B)** indicate that IDH-wt LGG was associated with longer overall survival (P < 0.001) and longer progression-free survival (P < 0.001) than IDH-wt GBM.

To understand patient prognosis, we collected survival data from the enrolled cohort. The overall survival (OS) was 55 months for the IDH-wt LGG patients and only 14.83 months for the IDH-wt GBM patients (Hazard ratio, 0.3213; 95% CI of hazard ratio, 0.2543-0.4060; P < 0.001). The median progression-free survival (PFS) was 44 months for IDH-wt LGG and only 9.767 months for GBM (HR, 0.3535; 95% CI, 0.2826-0.4421; P < 0.001) (**Figure 1**). By Kaplan-Meier survival analysis, we found significant differences in survival between the two groups, even though the treatment strategy for IDH-wt LGG was less aggressive than that for IDH-wt GBM. We performed univariable Cox regression analysis on the patient prognostic data and found that in LGG, patients with MGMT methylation had a better prognosis (*P* < 0.001). The prognosis of LGG patients who did not undergo radiotherapy or chemotherapy was better (*P* = 0.038). In LGG, the prognosis of grade 2 glioma was significantly better than that of grade 3 glioma (*P* < 0.001). In GBM, patients without PTEN mutations had a better prognosis (*P* = 0.023). Intraoperative total tumor resection (*P* = 0.015), postoperative radiotherapy (*P* = 0.034), and postoperative acceptance with chemotherapy (*P* < 0.001) were associated with a better prognosis. Then we performed a multivariate analysis using the SPSS software and found that the prognostic factors were different between the two groups of patients. In LGG, the OS and PFS were mainly affected by MGMT methylation (*P* < 0.001), TERT promoter mutation (*P* = 0.005), TP53 (*P* = 0.002), and the onset age (*P* < 0.001). While in GBM, the OS and PFS were more likely to be affected by interoperative resection (*P* = 0.004), radiotherapy (*P* = 0.006), chemotherapy (*P* = 0.001), and onset age (*P* = 0.03).

### Gene Expression Analysis

**Figure 2:**
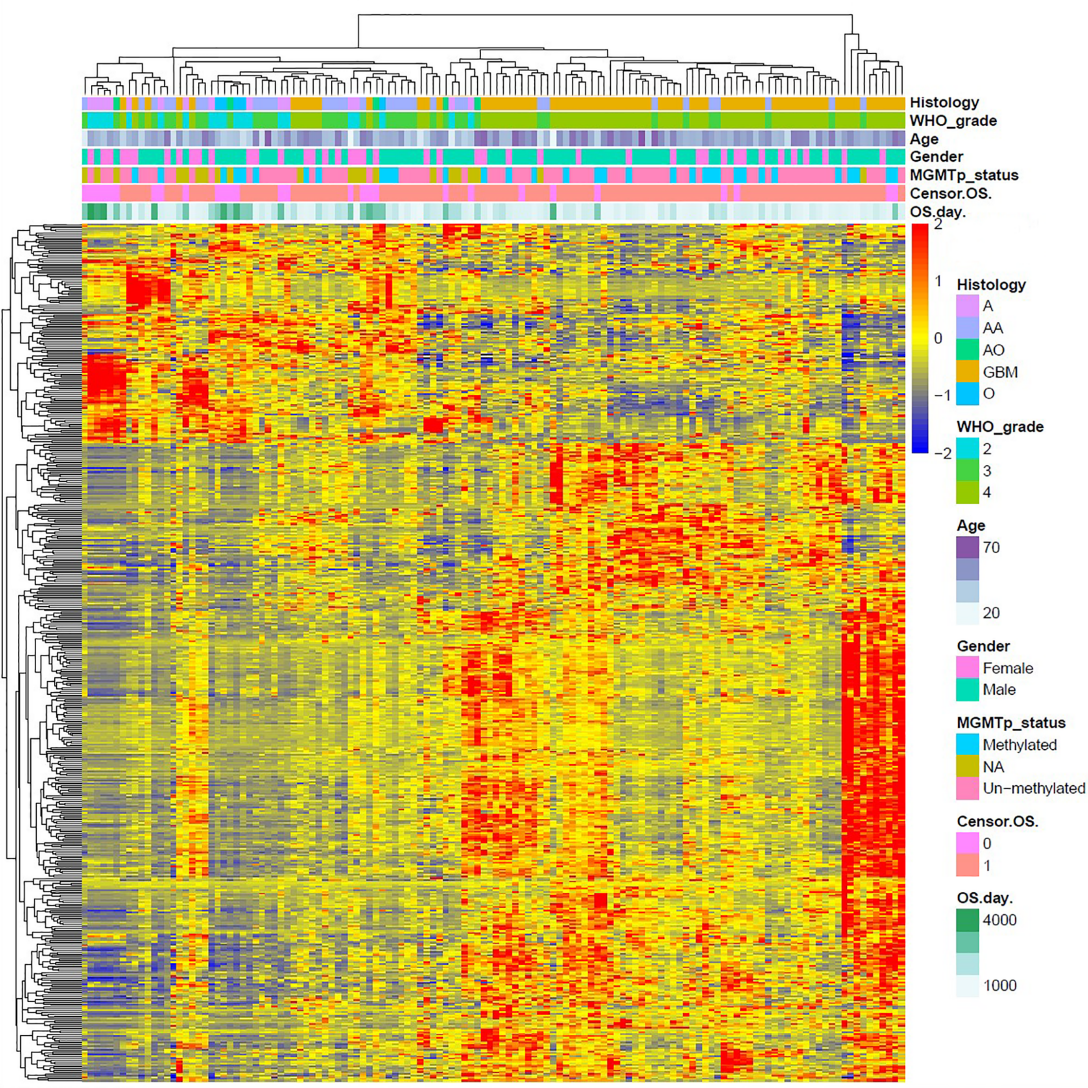
Supervised cluster analysis of the gene sequencing data. We obtained gene heat maps describing gene expression in the different patient groups and found that there were obvious differences in gene expression levels and gene expression types between IDH-wt LGG patients and IDH-wt GBM patients.

**Figure 3:**
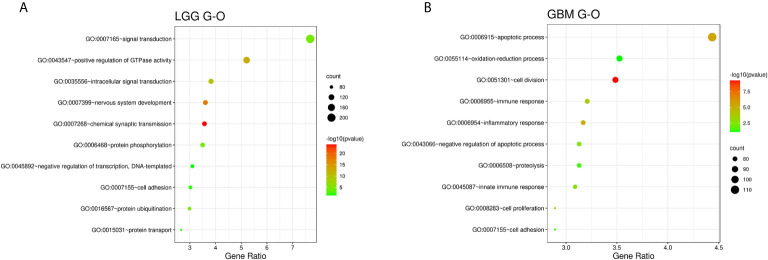
Functional enrichment analysis of associated genes for LGG **(A)** and GBM **(B)**, indicating the functional roles of the gene sets in the different subgroups. Enrichment results for biological processes were obtained from the GO database. The orders of the biological processes listed in the bubble chart are based on the number of targets annotated in biological process (BP).

We used R Studio to perform supervised cluster analysis on the 140 patient gene sequencing data (61 LGG and 79 GBM) and obtained gene heat maps describing the levels of gene expression in the different patient groups (**Figure 2**). Through the obtained gene heat map, it can be seen that there are obvious differences in the gene expression levels and gene expression types between IDH-wt LGG patients and IDH-wt GBM patients. Gene expression in LGG patients was more concentrated in the upper left corner of the picture, the first quarter of the list of genes, this part of the gene is low expressed in GBM patients. We screened the IDH-wt LGG and GBM genes from the database and performed Gene Ontology (GO) analysis. The results of the analysis were completely different for gliomas of different grades (**Figure 3**). The differentially expressed genes in patients with IDH-wt LGG were mainly enriched in the signal transduction and positive regulation of GTPase activity functions, whereas those in patients with IDH-wt GBM were mainly enriched in the functions of apoptotic process and oxidation–reduction process.

**Figure 4:**
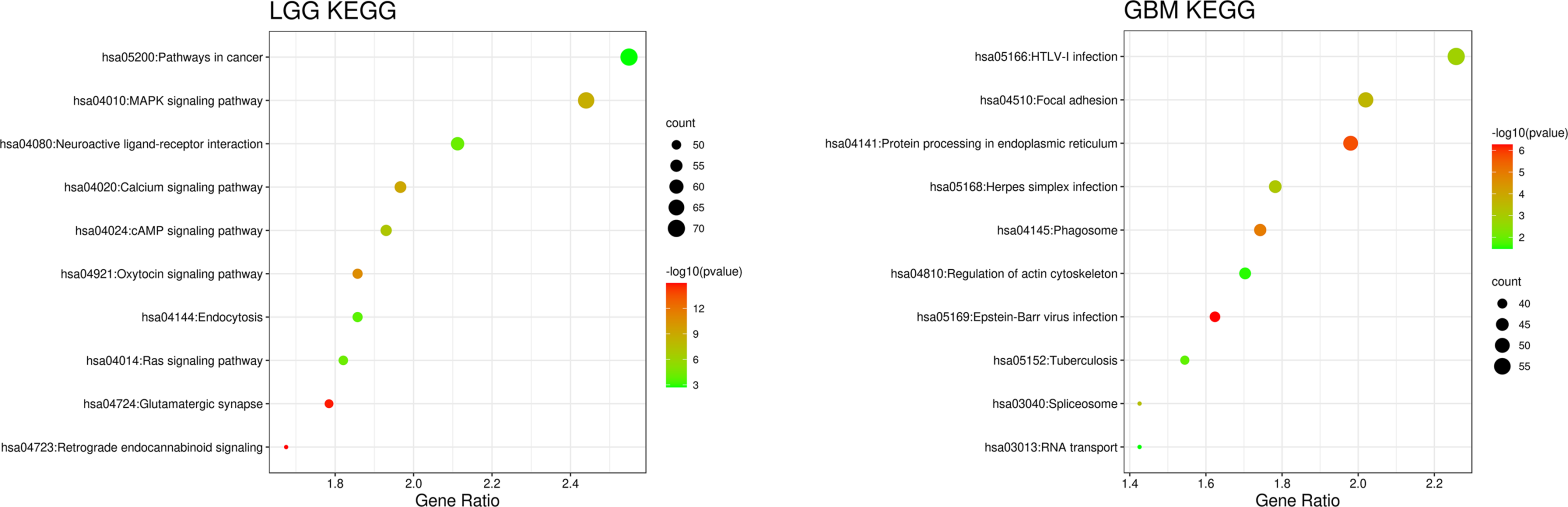
Patient gene expression analysis was performed using the Kyoto Encyclopedia of Genes and Genomes (KEGG) database provided by the DAVID website for correlation analysis. The orders of the biological processes listed in the bubble chart are based on the number of targets annotated in biological process (BP).

We compared the gene expression of the patients and used the Kyoto Encyclopedia of Genes and Genomes (KEGG) database provided by the DAVID website for correlation analysis and found that the cellular pathways of IDH wild-type LGG and GBM were completely different in terms of magnitude and function (21, 22). The differentially expressed genes in LGG were mainly enriched in metabolic pathways and MAPK signaling pathway, whereas those in GBM were mainly enriched in the Focal adhesion and HTLV-1 infection (**Figure 4**).

## Discussion

Following the release of the WHO’s new glioma classification in 2016, there has been a growing trend toward precision medicine. Our research compared the clinical information, molecular pathology, and gene expression between IDH-wt LGG and GBM patients. According to the results of our analysis, we found that there were significant differences between the two groups in the abovementioned aspects.

### Clinical Information

We use the chi-square test to analyze the two cohort patients, and we found that there is no significant difference between them in gender (*P* = 0.296). The IDH-wt LGG patients were more elderly than GBM patients in our patients (*P* < 0.01). According to Ostorm et al.’s research, GBM is mainly found in elderly patients (65-75), and LGG is mainly found in younger patients (age<65) (23). By comparing the two sets of data, we found that our conclusion is similar to them, but our patients have a younger onset age than them, this may be because our patients are all IDH-wt glioma patients, and these characteristics of molecular (IDH-wt) might lead to a tendency toward a younger age of onset in GBM, and the primary LGG patients was much more younger than the primary GBM patients. Epilepsy is one of the most common symptoms of glioma patients, so we collected the history of epilepsy of the enrolled patients. Through the chi-square test, we found that there was a significant difference between the two groups of patients (*P* < 0.001), so we believe that LGG patients are more likely to have epileptic through the course of the disease, which may because LGG is more incline to widespread invasion growth pattern, whereas the GBM are more likely show the characteristics of localized growth. Through the chi-square test, we found that there are no differences in the two patient groups in tumor location. In the treatment regimen, by collecting patient surgical data, we found that GBM patients are more inclined to undergo total tumor resection, and the LGG patients are more likely receive partial resection, which may because the course of LGG is longer and the scope of tumor invasion may be wider than GBM; furthermore, there is no obvious MRI enhancement boundary, so surgery for total resection may be difficult. Through the chi-square test, we found that there is a tendency that GBM patients were more likely to receive radiotherapy after the operations (*P* = 0.072). Besides, in LGG patients, 80 (48.5%) received TMZ for chemotherapy and 78 (47.3%) were not, whereas in GBM patients, 159 (71%) has TMZ for chemotherapy and 56 (25.0%) did not receive any chemotherapy. The chi-square test suggests that there have obviously been differences between them (*P* < 0.001). In summary, we concluded that there are significant differences between LGG and GBM in terms of clinical manifestations.

### Molecular Pathological Characteristics

Among the patients we collected, none of the LGG or GBM patients had 1p/19q codeletion. This result is consistent with the current major view (24). In LGG patients, eight (5.0%) patients have PTEN mutation and 26 (11.6%) GBM patients have PTEN mutation, which is more than double the rate for LGG, so we conducted that GBM are more incline to PTEN mutation (*P* = 0.010). By studying the previous literature, we found that the abovementioned data are consistent with the previously reported data (25–28). In regard to p53 mutational status and MGMT methylation status, we could not find any significant differences. The chi-square test results showed that the *P* value for the difference in P53 mutational status between IDH-wt LGG and GBM was 0.518, whereas that for the MGMT methylation status the *P* value was 0.52, indicating no statistically significant difference between the groups for either condition. And the same condition is true for TERT promoter (*P* = 0.991) and EGFR amplification (*P* = 0.204). Through the abovementioned analysis, we believe that although only some of the abovementioned molecular pathological characteristics are different between IDH-wt LGG and GBM, significant differences were demonstrated in at least PETN mutational status.

### Survival Analysis

By analyzing the survival data, we found that although the treatment strategy for IDH-wt LGG is less aggressive than GBM, its OS and PFS were still better (*P* < 0.001). It is generally accepted that some molecular characteristics are associated with a better prognosis, just like MGMT methylation and PTEN or P53. Combined with the molecular pathological characteristics, we believe that LGG is different from GBM. According to the research data from RTOG 9802, the OS and PFS of LGG are 7.5 and 4.4 years, respectively, which are significantly longer than what we reported. However, the RTOG 9802 was mainly focused on IDH mutation patients, whereas they found that the LGG patients have a better prognosis than GBM patients, and our study draws the same conclusion (29). Then we conducted a COX analysis and found a different conclusion about it. In LGG, the better prognosis is associated with MGMT methylation and no radiotherapy (*P* = 0.199) and no chemotherapy (*P* = 0.744). However, in GBM, the better prognosis is more inclined to NO PTEN mutation, total resection, radiotherapy, and chemotherapy. Besides, age affected both OS and PFS in LGG and GBM patients (**Table 2**).

**Table 2:** Multivariate analysis results for LGG and GBM patients.

		Event	B	P	OR	95% CR CI
LGG	OS	MGMT	1.239	<0.001	3.453	2.007–5.940
		TERT	−1.522	0.005	0.218	0.075–0.634
		TP53	1.142	0.002	3.134	1.542–6.446
		Radiotherapy	−1.538	0.199	0.215	0.021–2.247
		Chemotherapy	0.331	0.744	1.393	0.190–10.204
		Age	0.023	0.01	1.023	1.005–1.040
	PFS	MGMT	1.249	<0.001	3.487	2.076–5.856
		TERT	−0.687	0.006	0.503	0.307–0.824
		TP53	0.445	0.012	1.561	1.104–2.208
		Radiotherapy	−1.507	0.208	0.222	0.021–2.315
		Chemotherapy	0.474	0.64	1.606	0.220–11.731
		Age	0.029	<0.001	1.03	1.013–1.047
GBM	OS	MGMT	0.76	0.013	2.139	1.174–3.897
		PTEN	−0.709	0.038	0.492	0.252–0.961
		Resection	−0.575	0.004	0.562	0.277–1.141
		Radiotherapy	−0.402	0.006	0.669	0.190–2.360
		Chemotherapy	1.395	0.001	4.037	0.890–18.302
		Age	0.014	0.026	1.014	1.002–1.027
	PFS	Resection	−0.435	0.005	0.647	0.319–1.311
		Radiotherapy	−0.391	0.009	0.677	0.190–2.406
		Chemotherapy	1.626	0.033	5.084	1.139–22.692
		Age	0.014	0.03	1.014	1.001–1.026

### Gene Expression Analysis

**Figure 5:**
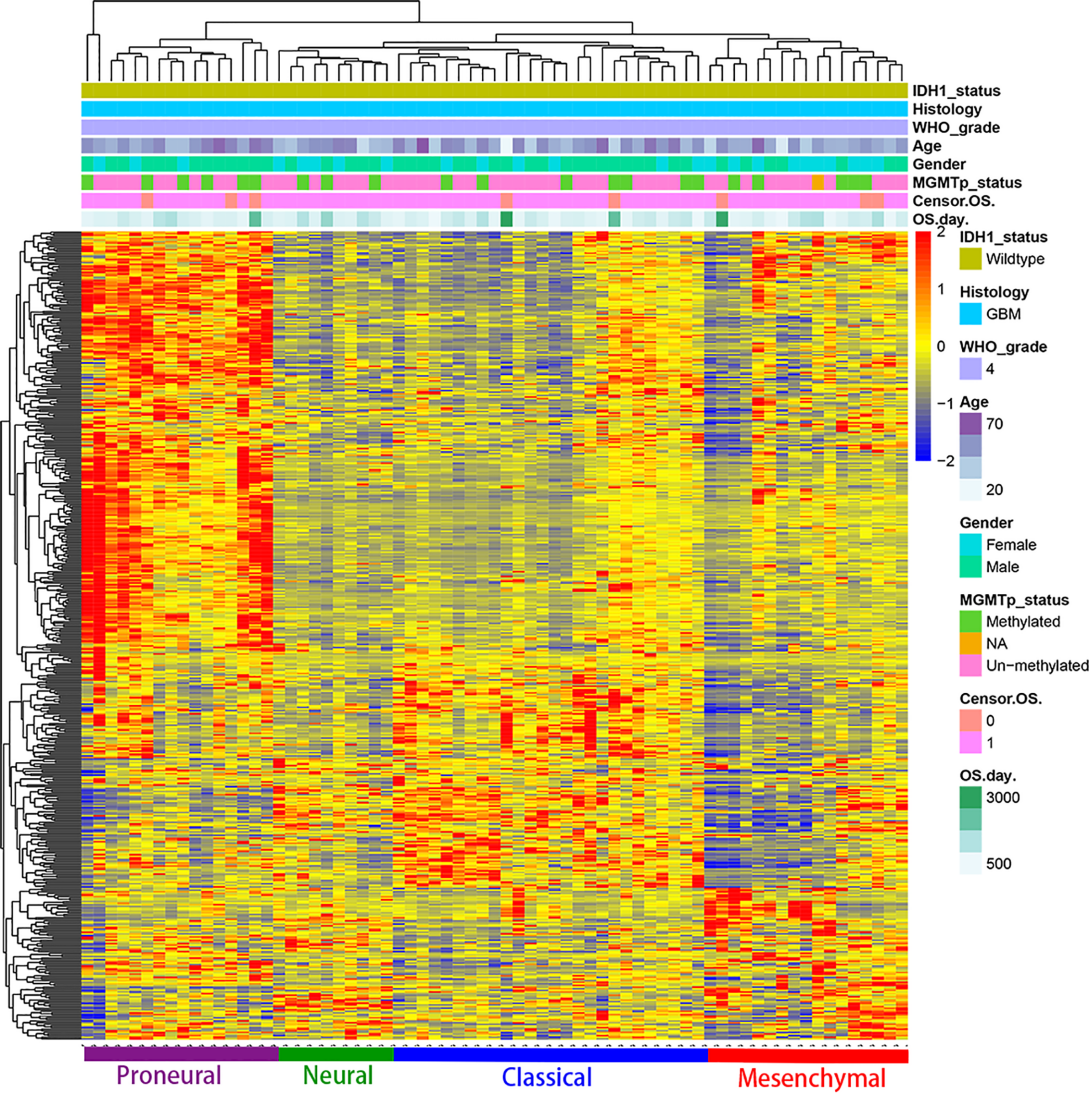
Supervised cluster analysis of gene expression in GBM patients shows it suits the Verhaak’s classes well.

Based on the above differences in clinical and molecular pathology, we found 3,000 differentially expressed genes from the CGGA database between the two groups through unpaired t-test analysis (*P* < 0.01). Through these differentially expressed genes, we conducted supervised clustering analysis and produced a gene heatmap. Through the heatmap, we found that the LGG patients were more concentrated in the first 700 genes. While the GBM was mainly gathering in the later 2000 genes. Since the GBM patients’ heatmap clearly split into different transcriptomic classes, we also conducted a supervised cluster analysis of gene expression in GBM patients alone and found that these patients met Verhaak’s classes (**Figure 5**) **(** 30). Then we used full genes list to perform GO analysis and KEGG pathway analysis and found that there were also differences in the signaling pathways and protein expression levels between LGG and GBM patients. The differentially expressed genes in LGG patients primarily performed the function of signal transduction (GO:0007165), positive regulation of GTPase activity (GO:0043547), and so on (**Figure 3A**), according to the G-O database, we found that LGG mainly focused on the upregulate signal transduction, activation of GTPase activity, and positive regulation of guanyl-nucleotide exchange factor activity, obviously, it could help the progress of the tumor, whereas those among GBM patients primarily performed the functions of apoptotic process (GO:0006915), oxidation-reduction process (GO:0055114), cell division (GO:0051301), and so on (**Figure 3B**). which means GBM has the function of positive regulation of apoptotic process and neuron apoptotic process. Besides, although the oxidation-reduction process is obsolete, it still shows that GBM has a hypermetabolic state, this might mean that GBM is in a highly proliferative state. Regarding to KEGG pathway analysis, the differentially expressed genes in LGG were more enriched in pathways in cancer (hsa05200), MAPK signaling pathway (has04010), and so on (**Figure 4A**), which was mainly associated with various cellular functions, including cell proliferation, differentiation, migration. Besides, it is also linked to evading apoptosis, proliferation, and sustained angiogenesis. All the pathways are associated with upregulating tumor growth, whereas those in GBM were more enriched in the HTLV-1 infection pathway (hsa05166), focal adhesion (hsa04510), and so on (**Figure 4B**). HTLV-1, which is human T-cell leukemia virus type 1 in full name, is a pathogenic retrovirus that is associated with adult T-cell leukemia/lymphoma (ATL) (31). However, it is also linked to some classical pathways and genes, such as PI3K-Akt and PTEN, so we believe it has a deep impact on GBM patients. Compared with LGG, the GBM G-O analysis and KEGG express were different.

Although IDH-wt LGG and GBM have many similarities, the current data and research are insufficient to show that they are the same, nor can IDH-wt LGG be treated the same way as GBM. Further research is needed to reclassify these diseases in greater detail. However, according to the recently released NCCN 2020 treatment guidelines, IDH-wt LGG should be treated more similarly to GBM. Therefore, whether this regimen is the most suitable treatment regimen should be further analyzed (13).

Although the results of our analysis are relatively significant, this study included data from the CGGA database, which contain data only on Asians, which may cause a certain bias in the gene expression. In addition, our study only included newly diagnosed patients and excluded recurrent GBM patients, which may also produce a certain bias for this latter group. Currently, treatment regimens for IDH-wt LGG in the NCCN treatment guidelines tend to be consistent with GBM, and the 3rd version of cIMPACT-NOW indicates that when IDH-wt diffuse astrocytoma has certain molecular pathological characteristics, it can be considered a WHO grade IV glioma; however, through our analysis of clinical information, molecular pathology, and gene expression, we found that there are still many differences between IDH-wt LGG and GBM (12, 13)

## Conclusion

Through the abovementioned analyses, we found that there are differences between LGG and GBM in terms of prognosis, epilepsy, resection range, PTEN mutational status, and biological behavior. These differences imply that whether the current treatment regimen is ideal still needs to be explored further.

## Data Availability Statement

Publicly available datasets were analyzed in this study. This data can be found here: Chinese Glioma Genome Atlas.

## Ethics Statement

The studies involving human participants were reviewed and approved by Ethics Committee of Beijing Tiantan Hospital. Written informed consent to participate in this study was provided by the participants’ legal guardian/next of kin.

## Author Contributions

PW was a major contributor in writing the manuscript. All authors contributed to the article and approved the submitted version.

## Funding

This work was supported by the following foundations. The funding sources had no influence on the design, performance, or reporting of this study. Beijing Municipal Bureau of Health (to XQ, Grant number: N/A), Beijing Natural Science Foundation (to YL, Grant number: 7192057), Beijing Hospitals Authority Youth Programme (to YL, Grant number: QML20190506).

## Conflict of Interest

The authors declare that the research was conducted in the absence of any commercial or financial relationships that could be construed as a potential conflict of interest.

The reviewer CY declared a shared affiliation, with no collaboration, with one of the authors, XQ, to the handling editor.
